# Energy intake, nutritional status and weight reduction in patients one year after laparoscopic sleeve gastrectomy

**DOI:** 10.1186/2193-1801-2-352

**Published:** 2013-07-30

**Authors:** Hanne Rosendahl Gjessing, Hans Jørgen Nielsen, Gunnar Mellgren, Oddrun Anita Gudbrandsen

**Affiliations:** Department of Clinical Science, University of Bergen, Bergen, Norway; Voss Hospital, Voss, Norway; Hormone Laboratory, Haukeland University Hospital, Bergen, Norway

**Keywords:** Obesity, Excess weight, Vitamins, Glucose, Insulin, Cholesterol, G0061stric sleeve

## Abstract

**Background:**

Laparoscopic sleeve gastrectomy (LSG) is increasingly popular due to its efficiency in reducing excess weight, however little is known about the nutritional status in patients after surgery.

**Purpose:**

To investigate how LSG affects energy intake, nutritional status and body weight one year after surgery.

**Methods:**

A total of 150 patients (116 women) were enrolled in the study. Data on body weight, waist circumference and blood samples were registered preoperatively and after surgery. Food intake was reported 3 and 12 months postoperatively.

**Results:**

The preoperative median BMI was 44.3 (inter quartile range 41.4-47.1), and was significantly reduced to 35.4 (32.6-38.6) after 3 months and further reduced to 30.5 (27.4-33.8) 12 months after surgery (p < 0.05). The median post surgery daily energy intake was significantly increased from 2971 (1982–3687) kJ after three months to 3840 (3046–4625) kJ twelve months postoperatively. One year after surgery, serum levels of folate, cobalamin, PTH and HDL cholesterol were significantly increased, whereas calcium, albumin, haemoglobin, creatinine, uric acid, CRP, glucose, insulin, insulin c-peptide, HOMA-IR, HbA1c and triacylglycerol were significantly decreased. Serum levels of vitamins E and D were unchanged after one year. The prevalence of patients with medically regulated type 2 diabetes was significantly reduced one year post surgery however no changes were seen in the prevalence of patients taking lipid lowering drugs or thyroxin.

**Conclusion:**

Based on the data obtained 12 months after surgery, LSG appears to be an effective treatment of morbid obesity without worsening the nutritional status despite the very low energy intake.

## Introduction

The prevalence of obesity has increased in recent decades, and obesity is now one of the leading public health concerns on a worldwide scale. Obesity causes or exacerbates many health problems, both independently and in association with other diseases. In particular, obesity is associated with the development of diabetes mellitus type 2, coronary heart disease, an increased incidence of certain forms of cancer, respiratory complications and osteoarthritis (Kopelman 2000).

During laparoscopic sleeve gastrectomy (LSG), about 90% of the stomach is surgically removed by vertical resection of the major side, including most of the fundus. The simplicity of the LSG surgical procedure, the low occurrence of complications, and the efficiency of reducing excess weight have made this restrictive procedure a popular choice for weight reducing surgery (Aurora et al. 2011; Nocca et al. 2008; Bohdjalian et al. 2010; Gluck et al. 2011; Eid et al. 2012). After LSG, the amount of food eaten is drastically reduced, potentially leading to nutritional deficiencies.

Very little is known about the nutritional status in patients after LSG, however, a few reports suggests that LSG results in fewer nutritional deficiencies compared to roux-en-Y gastric bypass (Toh et al. 2009; Gehrer et al. 2010). The aim of the present study was to investigate how LSG affects energy intake, weight loss and nutritional status 3 and 12 months after surgery.

## Methods

Patients submitted for LSG at Voss Hospital (Hordaland County, Norway) between September 2008 and May 2010 were included in the present study. This study was conducted according to the guidelines laid down in the Declaration of Helsinki and all procedures involving patients were approved by the Voss Hospital board as part of a quality assurance project. Thus, this paper is based on retrospective data, i.e. information and results that were collected routinely from patients in the clinic. Written informed consent was obtained from all patients. Participants were morbidly obese patients aged 18–60 years with BMI ≥ 40, or BMI ≥ 35 with comorbidities such as diabetes mellitus type 2 (DM2), sleep apnoea, or hypertension. The preoperative population included 150 patients (116 women).

Examinations were conducted 2–4 months prior to surgery, and 3 and 12 months after surgery. Weight, height and waist circumference were measured and fasting blood samples were drawn at all visits. All patients were seen by a dietician for routine dietary assessment and counselling at all study visits.

The preoperative protocol involved a 4.2 MJ/day (1000 kcal) diet supplemented with vitamins and long chain omega-3 fatty acids for the last 2 months preoperatively with the purpose for the patients to lose weight (5–10 kg), reduce fatty liver, and to decrease the risk of complications associated with surgery (Benotti et al. 2009). The patients were given recipes for suggestions on how to prepare all meals (breakfast, lunch, dinner, evening meal and fruits between meals), and were instructed to drink 1.5 L of water, sugar free lemonade, coffee, tea or bouillon daily, and to stop smoking.

During the LSG procedure, a narrow gastric tube was constructed along the minor side of the stomach, around an intragastric 32-French bougie, giving the patients a stomach volume of about 100 mL. A methylene blue test was performed peroperatively to exclude staple-line leakage. An x-ray contrast study was performed on the second postoperative day, and oral fluids were allowed when no leakage was demonstrated.

Postoperatively, patients were recommended to only drink clear fluids (water, bouillon, or ProvideXtra from Fresenius Kabi, Norway) for the first 3 days. On days 3–21 the patients follow a liquid diet with two protein rich nutritional supplements and fluids mixed with Resource Meritene (Nestle Nutrition, Norway). After 3 weeks, the patients were advised to eat mashed food for one week and then gradually to start eating small portions of protein rich food. Patients were given Somac Control (Takeda Nycomed), 20 mg/day for 100 days postoperatively as acid reducing medication. The patients were instructed to take supplements of multivitamins (5 ug vitamin D/day, Vitamineral, Vitaplex), long chain omega-3 fatty acids containing vitamin D (10 ug vitamin D/day, Møllers Dobbel, Møllers) and calcium citrate (500 mg calcium/day, Solaray Calcium Citrate, Solaray).

### Analyses of serum and blood samples

Vitamins, minerals, lipids and proteins levels were measured in serum samples, and hemoglobin and HbA1c (glycated hemoglobin A1c) was measured in whole blood added EDTA, performed by routine methods at the Hormone Laboratory at Haukeland University Hospital (Bergen, Norway) and at the Laboratory at Voss Hospital (Voss, Norway). The insulin sensitivity (HOMA-IR)(Matthews et al. 1985) was calculated from fasting plasma glucose and insulin concentrations as glucose [mmol/L] × insulin [mU/L] divided by 22.5.

### Estimation of energy intake

Twenty-four hour recall interviews were conducted 3 and 12 months postoperatively to estimate the patients’ energy intake. Calculations were performed using “Mat på Data” version 5.1 (The Norwegian Food Safety Authority, the Directorate for Health and the Department of Nutrition at the University of Oslo).

### Statistical analysis

Analysis of data was performed using PASW 19.0 software (IBM Corporation, NY), with statistical significance set at p < 0.05. The distribution of the data was tested using Q-Q-plots, and since most data were non-parametric distributed, differences in the nutritional parameters of the patients before and after surgery were investigated using Wilcoxon Signed Ranks Test for nonparametric distributions. McNemar Test was used to compare the prevalence of abnormal and normal levels before and after surgery, using reference values according to the Hormone Laboratory at Haukeland University Hospital (Bergen, Norway) and the Laboratory at Voss Hospital (Voss, Norway). Correlation between vitamin D and PTH was assessed by using Spearman's rho test.

## Results

One-hundred and fifty patients (116 women) submitted for LSG were enrolled in the study, with a median age of 43 years (intequartile range 34–51) (Table 1). Preoperative bodyweight, BMI, percent excess body weight and waist circumference (WC) is presented in Table 1. Of these 150 patients, 139 patients (107 women) completed the examination 3 months post surgery, and 125 patients (92 women) completed the examination 12 months post surgery.

**Table 1:** **Preoperative patient characteristics**

	Median	Interquartile range
Men/women		34/116
Age (y) ^1^	43	34–51
Bodyweight (kg) ^1^	126.0	115.3–139.8
BMI (kg/m2) ^1^	44.3	41.4–47.7
Excess body weight (%)^1^	43.6	39.5–47.6

^1^Values as median (interquartile range).

The preoperative median BMI and WC for all patients were significantly reduced after three months and further reduced after twelve months (Table 2). Correspondingly, the percent excess weight loss (%EWL) after surgery was increased from 3 to 12 months post surgery. Estimation of energy intake from 24 h recall interviews show that the daily energy intake was very low after 3 months (2971 kJ, or 710 kcal) but was significantly increased 12 months postoperatively (3840 kJ, or 918 kcal). No data was available for energy intake before surgery.

**Table 2:** **BMI, waist circumference, excess body weight loss and energy intake preoperatively and 3 and 12 months after LSG**

	Preoperative		3 months postoperative		12 months postoperative	
	(N = 150)		(N = 139)		(N = 125)	
	Median	Interquartile range	Median	Interquartile range	Median	Interquartile range
BMI, kg/m2	44.3	41.4–47.1	35.4^*^	32.6–38.6	30.5^*,**^	27.4–33.8
WC^1^, cm	131	123–138	112^*^	105–121	100^*,**^	94–108
EWL^2^, %	0		45.7	38.1–55.6	72.1^**^	58.5–86.7
EI ^3^, kJ/day	NA		2971	1982–3687	3840^**^	3046–4625

^1^N = 138 preoperatively, N = 105 three months postoperatively, N = 118 twelve months postoperatively.

^2^based on excess weight as starting at BMI > 25 (Almogy et al. 2004).

^3^N = 107 three months postoperatively, N = 120 twelve months postoperatively.

Wilcoxon Signed-Rank Test for pair wise comparison was used to investigate changes over time.

^*^significantly different from preoperatively (p < 0.001).

^**^significantly different from 3 months postoperatively (p < 0.001).

*WC* waist circumference, *EWL* excess body weight loss, *EI* energy intake, *NA* not available.

The median serum level of calcium was decreased after 12 months when compared to baseline (Table 3). No change was seen in the prevalence of patients with calcium level in the normal reference range when pre and post surgery levels were compared. The serum folate and cobalamin (vitamin B12) levels were increased, whereas no changes were seen in levels of vitamins E and D, and 1,25-dihydroxy vitamin D, after one year (Table 3). The prevalence of patients with folate below reference range was significantly decreased from pre surgery to 12 months post surgery, whereas no changes were seen in the prevalence of patients with vitamin levels in the normal reference range for cobalamin, vitamin E, vitamin D and 1,25-dihydroxy vitamin D.

**Table 3:** **Serum concentrations and prevalence**^$^**(under/in/above reference range) of calcium and vitamins preoperatively and 12 months after LSG**

	Preoperative (N = 150)			12 months postoperative (N = 125)		
	Median	Inter quartile range	Prevalence (%)	Median	Inter quartile range	Prevalence (%)
Calcium, mmol/L	2.36	2.29–2.41	5/92/3	2.33^*^	2.27–2.40	4/95/1
Folate^1^, nmol/L	11.3	8.2–15.8	23/77	16.0^**^	10.7–23.0	8/92†
Cobalamin , pmol/L	265	202–323	16/82/2	278^*^	209–421	8/86/6
Vitamin E^2^, umol/L	23.8	20.3–28.7	3/91/6	23.7	19.9–27.7	1/97/2
25-OH-vitamin D^3^, pmol/L	50.1	36.3–65.0	47/52/1	50.0	35.3–68.9	49/51/0
1,25-di-OH-vitamin D^4^	84	67–104	8/89/3	84	68–102	3/96/1

^1^N = 119 twelve months postoperatively, ^2^ N = 120 twelve months postoperatively, ^3^ N = 117 twelve months postoperatively, ^4^ N = 141 preoperatively, N = 111 twelve months postoperatively.

^$^Reference ranges used: Calcium 2.20-2.55 mmol/L; Folate > 8 nmol/L; Cobalamin 175–700 pmol/L; Vitamin E 11.6-40.0 umol/L; 25-hydroxyvitamin D 50–150 pmol/L; 1,25-dihydroxy-vitamin D 50–150 pmol/L.

Wilcoxon Signed-Rank Test for pair wise comparison was used to investigate changes over time.

^*^significantly different from preoperatively (p < 0.05).

^**^significantly different from preoperatively (p < 0.001).

McNemar Test was used to compare the prevalence of values below normal reference range between pre and post surgery values.

†significantly different from preoperatively (p < 0.005).

The serum albumin level was significantly decreased after 3 months and stayed decreased after 12 months, with no change in the distribution of normal and abnormal values post surgery (Table 4). Haemoglobin and creatinine serum levels were decreased after 3 months, and further decreased after 12 months. The prevalence of haemoglobin levels below normal range was increased after three months but was comparable to pre surgery values after 12 months, whereas no change in prevalence of normal levels was seen for creatinine. Uric acid level was decreased after 12 months concomitant with marked reduction in the prevalence of patients with uric acid levels above reference range. The median PTH level was increased after 12 months, but the prevalence of patients with PTH levels in the normal reference range was unchanged. The CRP serum level and the prevalence of abnormally high CRP levels were reduced after 3 months, and both were further reduced after 12 months.

**Table 4:** **Serum concentrations and prevalence**^$^**(under/in/above reference range) of albumin, haemoglobin, creatinine, uric acid, PTH and CRP preoperatively and 3 and 12 months after LSG**

	Preoperative (N = 150)			3 months postoperative (N = 139)			12 months postoperative (N = 125)		
	Median	Inter quartile range	Prevalence (%)	Median	Inter quartile range	Prevalence (%)	Median	Inter quartile range	Prevalence (%)
Albumin^1^, g/L	44	43–46	1/78/21	43^**^	41–45	2/83/15	43^**^	42–45	0/91/9
Haemoglobin, g/dL	14.1	13.4–14.9	1/98/1	13.9^*^	13.0–14.7	7/89/4†	13.7^**,***^	13.0–14.4	5/94/1
Creatinine, umol/L	65	57–72	6/91/3	63^*^	56–70	4/95/1	60^**,****^	55–66	10/89/1
Uric acid, umol/L	354	305–421	0/99/51	NA			284^**^	248–340	1/92/7†
PTH, pmol/L	4.1	3.0–6.1	2/80/18	NA			5.0^*^	3.5–6.7	2/77/21
CRP^2^, mg/L	6.0	4.0–11.0	37/63	4.0^*^	4.0–8.0	57/43 †	4.0^**,****^	4.0–4.0	90/10†‡

^1^N = 105 three months postoperatively, ^2^ N = 142 preoperatively, N = 105 three months postoperatively.

^$^Reference ranges used: Albumin 18–39 yr: 36–48 g/L, 40–69 yr: 36–45 g/L; Hemoglobin F ≥ 18 yr: 11.7-15.3 g/dL, M ≥ 18 yr: 13.4-17.0 g/dL; Creatinine F: 45–92 umol/L, M:60–105 umol/L; Uric acid F < 50 yr: 155–350 umol/L, F > 50 yr: 155–400 umol/L, M:230–480 umol/L; PTH 1.3-6.8 pmol/L; CRP <5 mg/L.

Wilcoxon Signed-Rank Test for pair wise comparison was used to investigate changes over time.

^*^significantly different from preoperatively (p < 0.05).

^**^significantly different from preoperatively (p < 0.001).

^***^significantly different from 3 months postoperatively (p < 0.05).

^****^significantly different from 3 months postoperatively (p < 0.001).

*NA* not available.

McNemar Test was used to compare the prevalence of values below normal reference range between pre and post surgery values.

†significantly different from preoperatively (p < 0.005).

‡ significantly different from 3 months postoperatively (p < 0.005).

The median levels of fasting glucose, insulin, insulin c-peptide, HOMA-IR and HbA1c levels were significantly reduced after 12 months (Table 5). The prevalence of patients with elevated fasting glucose level was significantly decreased and there was a decreased prevalence of patients with insulin in the normal range whereas prevalence of insulin c-peptide in normal range was unchanged. Also, a significant increase was seen in the number of patients with HOMA-IR below 2 and of patients with HbA1c level in the normal range.

**Table 5:** **Circulating levels and prevalence**^$^**(under/in/above reference range) of glucose, insulin, HOMA-IR, insulin c-peptide, lipids, and HbA1c preoperatively and 12 months after LSG**

	Preoperative			12 months postoperative		
	(N = 150)			(N = 125)		
	Median	Inter quartile range	Prevalence (%)	Median	Inter quartile range	Prevalence (%)
Glucose^1^, mmol/L	5.5	5.2–6.0	1/76/23	4.9^**^	4.7–5.3	1/92/7†
Insulin^2^, mU/L	9.0	6.1–13.7	24/74/2	3.3^*^	2.0–6.7	73/27/0†
Insulin c-peptide^3^, nmol/L	0.94	0.69–1.18	3/92/5	0.61^*^	0.51–0.84	10/90/0
HOMA-IR^A4^	2.3	1.5–3.4	39/42/19	0.7^*^	0.5–1.4	85/12/3†
HbA1c^**B**5^, %	5.7	5.4–6.1	0/83/17	5.2^*^	5.0–5.5	0/96/4†
Total cholesterol^6^, mmol/L	5.0	4.4–5.8	6/84/10	5.0	4.4–5.6	2/95/3
HDL cholesterol^7^ , mmol/L	0.9	0.8–1.1	47/53/0	1.5^*^	1.3–1.7	2/98/0†
Triacylglycerol, mmol/L	1.97	1.31–2.61	1/75/24	0.95^*^	0.75–1.33	1/98/1†

^1^N = 137 preoperatively, ^2^ N = 145 preoperatively, ^3^ N = 146 preoperatively, ^4^ N = 135 preoperatively, ^5^ N = 133 preoperatively.

^6^N = 121 twelve months postoperatively, ^7^ N = 131 preoperatively.

^$^Reference ranges used: Glucose 4.0-6.0 mmol/L; Insulin 6.0-27.0 mU/L; Insulin c-peptide 0.4-1.7 nmol/L; HOMA-IR <2 nmol as normal value, ≥ 2 nmol/L is pathological, > 4 reflecting the pre-diabetic stage; HbA1c 4.0-6.4%; Total cholesterol 18–29 yr: 2.9-6.1 mmol/L, 30–49 yr:3.3-6.9 mmol/L , ≥50 yr: 3.9-7.8 mmol/L; HDL cholesterol F: 1.0-2.7 mmol/L, M:0.8-2.1 mmol/L; Triacylglycerol 0.45-2.60 mmol/L.

Wilcoxon Signed-Rank Test for pair wise comparison was used to investigate changes over time.

^*^significantly different from preoperatively (p < 0.001).

*NA* not available.

^A^Homeostasis model assessment – insulin resistance, prevalence shown as normal/pathological/pre-diabetic stage.

^B^Glycated hemoglobin A1c.

McNemar Test was used to compare the prevalence of values below normal reference range between pre and post surgery values.

†significantly different from preoperatively (p < 0.005).

‡significantly different from preoperatively (p < 0.05).

No change was seen in levels or prevalence of abnormal of total cholesterol level in patients one year after surgery. However, the HDL cholesterol level was significantly increased and triacylglycerol level was significantly decreased after one year, whereas the prevalences of abnormal levels of both HDL cholesterol and triacylglycerol were significantly decreased.

Only one patient with type 1 diabetes was enrolled in the study, and this patient did not complete the 12 month postoperative visit (Table 6). A significant reduction in the use of antidiabetics was seen 12 months after surgery, and concomitantly there was a significantly lower prevalence of type 2 diabetes one year post surgery. No significant changes were seen in the prevalence of patients taking lipid lowering drugs or thyroxin.

**Table 6:** **Prevalence of diabetics and use of lipid lowering drugs or thyroxin preoperatively and 12 months after LSG**

	Preoperative	12 months postoperative
Type 1 diabetes	1%	^$^
Type 2 diabetes, taking insulin	1%	2%
Type 2 diabetes, medicated	14%	1%†
Type 2 diabetes, dietary regulated	1%	3%
No diabetes	83%	94%†
Lipid lowering drugs	20%	13%
Thyroxin users	14%	15%

McNemar Test was used to compare the prevalence of values below normal reference range between pre and post surgery values.

^$^One patient with type 1 diabetes was enrolled in the study, and this patient did not complete the 12 month postoperative visit.

†significantly different from preoperatively (p < 0.05).

## Discussion

The preoperative and 3 and 12 months postoperative nutritional status and weight reduction were investigated in 150 patients submitted for LSG surgery. As anticipated, BMI was significantly reduced 3 and 12 months postoperatively. The %EWL 12 months post surgery was reduced by 72.1, which is comparable to findings reported by others for patients with similar pre surgery BMI (Brethauer et al. 2009). Correspondingly, WC was significantly reduced after surgery, although the mean WC one year after surgery for 78% of our patients was still above the action level by WHO set at ≥102 cm for men and ≥88 cm for women (WHO 1997).

The median energy intake 3 months after surgery was very low (2971 kJ/day), but was significantly increased after 12 months (3840 kJ/day), which is an important finding as very little is known about the caloric intake after LSG. Our findings are comparable to findings in a small study (N = 21) reporting a median intake of 3266 kJ/day three months after longitudinal gastrectomy (Almogy et al. 2004). The increased energy intake over time in the present study may be explained by an improved food tolerance (Overs et al. 2012), intake of more energy dense food and probably also a certain moderate widening of the gastric tube.

The low energy intake the first years post surgery is challenging for the LSG patients as they need both a detailed dietary plan and dietary supplements to achieve sufficient intake of macro and micro nutrients, since it is difficult to meet the daily requirements of vitamins and minerals with a daily energy intake below 8.4 MJ (FAO/WHO 2001). Also, the removal of most of the fundus and hence the reduction in parietal cells, in addition to nausea, vomiting and food intolerance experienced by some put LSG patients at high risk for nutrient deficiencies. It has been suggested that LSG has little impact on macronutrient deficiency since the procedure is restrictive and therefore does not alter the absorption in the small intestine (Baltasar et al. 2005). However, after LSG the stomach contents may empty more rapidly into the small intestine (Melissas et al. 2007), which in turn may reduce the bioavailability and absorption of nutrients. Although the median albumin level was decreased, the serum albumin level was normal in the majority of our patients both preoperatively and postoperatively, and thus one can assume that in general the patients had an overall sufficient protein intake after one year, which is in line with findings by others (Damms-Machado et al. 2012; Hamoui et al. 2006).

Generally, LSG is believed not to cause fat malabsorption and thus the absorption of fat soluble vitamins should not be affected. Vitamin E deficiency was observed in four patients before and in only one patient one year after surgery, and median levels were similar at these time points. The prevalence of patients with vitamin D deficiency was high (47%) possibly caused by reduced bioavailability of vitamin D due to sequestration by adipose tissue (Wortsman et al. 2000). However, neither the median vitamin D level nor the prevalence of patients with vitamin D within the reference range were changed as patients lost weight. The patients were advised to take vitamin D supplements, but due to the high prevalence of vitamin D deficiency one can assume a low compliance or low bioavailability as has been observed by others after LSG (Capoccia et al. 2012). It is essential that the vitamin D level is within the normal range for sufficient intestinal calcium uptake, and a lack of vitamin D will lead to a negative calcium balance and thus change bone metabolism, and may cause a compensatory rise in PTH to promote bone resorption (Aarts et al. 2011). Hyperparathyroidism has been observed in a large proportion (25-48%) of morbidly obese subjects (Hamoui et al. 2004; Carlin et al. 2006), and in the present study 18% of the patients had PTH above normal range. After one year, the median PTH level was significantly increased without affecting the prevalence of patients with PTH above reference range, and the vitamin D and PTH levels were significant inversely correlated (p < 0.01) both before and one year after surgery. The reduction in calcium level, despite the patients were instructed to take calcium supplements, is probably caused by the reduced intake of calcium containing food since these patients have a low energy intake after surgery, whereas the unchanged vitamin D level is likely due to patients taking supplements as food itself is a less important source for this vitamin in this population.

The absorption of cobalamin from food is dependent on the presence of intrinsic factor and HCl, therefore the resection of fundus may result in cobalamin deficiency postoperatively if patients are not treated with oral supplementations or injections of cobalamin (Capoccia et al. 2012). Bariatric patients struggle with the digestion of protein rich food in particular red meat (Elliot 2003) which is a good source of cobalamin, and the protein intake is reported to be below the recommended level for a high proportion of patients after LSG (Andreu et al. 2010). Despite that, an increased cobalamin level was observed one year after surgery, possibly due to patients' use of cobalamin supplements or injections as advised by health personnel after LSG, however the prevalence of abnormal cobalamin levels was not significantly changed. This is in contrast to findings by others (Hakeam et al. 2009) reporting an increased prevalence of low cobalamin levels one year after LSG. Folate deficiency is not common after LSG, since this vitamin can be absorbed throughout the intestine, and therefore the observed low folate level post surgery is probably a result of low intake of food rich in this vitamin, such as legumes and green leafy vegetables, and not due to reduced intestinal uptake. A significant decrease in the prevalence of abnormal folate level from before surgery was observed in the present study, and folate deficiency was present in only 8% of the patients 12 months after surgery which is comparable with findings by others (Hakeam et al. 2009).

Obese patients usually have a chronic low-grade inflammatory condition, and this may affect many of the biochemical and hormonal processes in the body, and it has been have shown that LSG may improve the immune status (Hakeam et al. 2009; Esposito et al. 2003). Before surgery, about one third of our patients had elevated CRP levels, and one year after surgery this prevalence was markedly reduced to 10%, which is line with finding by others (Hakeam et al. 2009). Concomitant with lower CRP level, the reduced serum uric acid level also suggests an improvement of the inflammatory status (Gagliardi et al. 2009) in these patients.

Several studies confirm the efficiency of LSG in treatment of diabetes mellitus type 2 in morbidly obese patients (Rosenthal et al. 2009; Lee et al. 2010). One year after LSG, the glycemic control was significantly improved, with lower fasting glucose, insulin, insulin c-peptide, HOMA-IR and HbA1c and increased prevalence of patients within the normal range of these parameters except for insulin c-peptide, probably as a result of weight loss. Concomitant with this, a significant reduction in the number of patients with medically regulated diabetes and lower prevalence of type 2 diabetes were seen 12 months after surgery.

As an indirect measure of the improved nutritional status, and probably as a result of weight loss in the patients, the circulating levels of HDL cholesterol was significantly increased and that of triacylglycerol was significantly decreased, which is in line with findings by others (Benaiges et al. 2012). All patients had HDL levels above of 0.9 mmol/L one year after surgery, which is the threshold level defined by WHO (WHO 1999) for HDL cholesterol for metabolic syndrome, thus these findings suggests that our patients had a reduced risk of developing this syndrome one year after surgery.

There are some limitations to this study. First, not all patients completed medical examinations after 3 or 12 months, and we have no information about why these did not complete the protocol. Second, obese patients are known to under-report their dietary intake (Lissner et al. 2000) and therefore the estimated energy intake based on the 24 h recall interviews may be lower than the actual intake.

In conclusion, based on the data obtained 12 months after surgery, LSG has significant effect on weight loss and appears to be an effective treatment of morbid obesity, probably mediated through low energy intake. Despite the low energy intake, the patients' nutritional status did not worsen one year after surgery. The long term effect on weight loss, energy intake and nutritional status of the LSG procedure should be further investigated.
